# Shallow-Tapered Chirped Fiber Bragg Grating Sensors for Dual Refractive Index and Temperature Sensing

**DOI:** 10.3390/s21113635

**Published:** 2021-05-24

**Authors:** Takhmina Ayupova, Madina Shaimerdenova, Daniele Tosi

**Affiliations:** 1 School of Engineering and Digital Sciences, Nazarbayev University, Nur-Sultan 010000, Kazakhstan; takhmina.ayupova@nu.edu.kz (T.A.); madina.shaimerdenova@nu.edu.kz (M.S.); 2 Laboratory of Biosensors and Bioinstruments, National Laboratory Astana, Nur-Sultan 010000, Kazakhstan

**Keywords:** optical fiber sensors, fiber tapers, fiber Bragg gratings, chirped fiber Bragg grating, refractive index sensor, dual sensing

## Abstract

In this work, we present a gold-coated shallow-tapered chirped fiber Bragg grating (stCFBG) for dual refractive index (RI) and temperature sensing. The stCFBG has been fabricated on a 15-mm long chirped FBG, by tapering a 7.29-mm region with a waist of 39 μm. The spectral analysis shows two distinct regions: a pre-taper region, in which the stCFBG is RI-independent and can be used to detect thermal changes, and a post-taper region, in which the reflectivity increases significantly when the RI increments. We estimate the RI and thermal sensitivities as 382.83 dB/RIU and 9.893 pm/°C, respectively. The cross-talk values are low (−1.54 × 10^−3^ dB/°C and 568.1 pm/RIU), which allows an almost ideal separation between RI and thermal characteristics. The stCFBG is a compact probe, suitable for long-term and temperature-compensated biosensing and detection of chemical analytes.

## 1. Introduction

Optical fiber sensors allow the measurement of physical and biological parameters with high precision and operate in a broad range of environments [1] thanks to their advantageous properties, such as compactness and lightweight form factors, possibility to measure in situ (for example, through packaging in medical devices [2]), immunity to electromagnetic interference which allows the operation during magnetic resonance procedures [3], and excellent safety.

Refractive index (RI) sensors find increasing applications in biosensors [4]. RI sensors usually operate in two typologies of environments. A bare RI sensor, or refractometer, is capable of detecting small changes of the analyte surrounding the fiber, by measuring a wavelength shift or intensity change of the reflection or transmission spectrum of the device [5]. In a second step, RI sensors can be functionalized to the selective detection of a biological analyte, targeting specific biomolecules or cells, such as in the case of immunosensors [6]. The biofunctionalization step, usually performed by a thin-film metallic layer or through silanization [7], enables a selective detection, rather than a simple inspection of the surrounding analyte.

Three main technologies have been consistently used as RI sensors. A first approach makes use of in-fiber surface plasmon resonance (SPR) [8], in which p-polarized light input to a thin-film coated large-core fiber excites plasmonic waves that vanish in the metallic overlay. SPR sensors are usually low-cost and highly sensitive, and have also been demonstrated on plastic fibers [9], U-bent fibers [10], and smartphone hardware systems [11]. A second approach, having a much narrower bandwidth and, therefore, compatible with infrared spectrometers and optical fiber interrogators, makes use of fiber Bragg gratings (FBGs) [4]. By properly modifying the structure of an FBG to enable the interrogation of the fiber surrounding environment, it is possible to achieve sensors with high sensitivity and figures of merit. FBG-based solutions that have been recently reported include etched FBGs [7], tilted FBGs [12] also combined with plasmonic effects [13], etched-tilted FBGs [14], and long-period gratings [15]. Finally, the third class of biosensors includes devices based on interferometry, which guarantees excellent sensitivity ratings, but also implies operating with a large sensor bandwidth. Several approaches have been reported using Fabry–Perot interferometry [16], microfiber interferometers [17], Sagnac loops [18], and microstructured interferometers [19].

An interesting approach for biosensing is the use of fiber tapers [20], which are fabricated by reducing the thickness of an optical fiber to enable a change of effective refractive index [21]. Unlike chemical etching, which reduces the thickness by depleting the outer cladding, tapers are fabricated through fusion methods and maintain the same proportions between the core and cladding of the fiber. Tapers are often used in optical tweezers and in fiber core adapters, but also for biosensors, by reducing the fiber thickness to a small fraction of the original size, to create an interferometric structure. Huang et al. [22] reported a taper with 7.5-μm thickness fabricated with a flame-heat technique, which acts as a fiber modal interferometer. Tian et al. [23] fabricated a concatenated two-taper taper structure whereas each taper acts as the mirror of an interferometer; the tapers have 40-μm thickness with a distance of 24–55 mm between them. Sun et al. [24] reported a label-free biosensor based on a fiber taper with 5-μm thickness based on a double-cladding fiber.

Overall, to achieve an interesting sensitivity rating through a fiber taper, it is necessary to either taper the fiber down to <8-μm thickness to obtain high evanescent power or to concatenate multiple tapers in cascade. This poses two orders of problems: at first, deeply tapered fibers, as well as similarly tapered structures, are extremely fragile as the mechanical strength of the fiber is deeply compromised, limiting the possibility to operate in situ, for example, through a medical catheterization, as the fiber can easily break. In addition, optical fiber splicers (either arc-fusion or based on a laser) that automate the fabrication of devices cannot handle tapers that reduce the diameters by a factor larger than 10 (nominally), which reduces to <7 experimentally. Considering that standard telecom fibers such as the SMF-28 have a cladding size of 125 μm, this means that only tapers with a diameter ≥18 μm can be fabricated through splicers that have high precision. In fact, most biosensors are based on heat fusion setups, which are known to have a poor throughput in terms of the number of tapers successfully achieved with respect to the actual fabricated number.

In this work, we propose a different approach that rewards the ease of fabrication and interrogation of the sensor, as well as its possible operation. We report the design of a shallow-tapered sensor (taper waist diameter of 39 μm) fabricated on a pre-existent chirped FBG (CFBG) [25]. The structure achieves a spectrum that clearly identifies two pre-tapered and post-tapered spectral slices, the latter varying in intensity when exposed to RI changes. In addition, the structure guarantees a separate temperature measurement, by detecting the wavelength shift of the whole spectrum. We report a sample having a taper waist diameter of 39 μm, enabling dual RI–temperature sensing. 

The fabrication of the proposed shallow-tapered CFBG (stCFBG) is much simpler than other tapered fiber RI sensors and biosensors, as it is a one-step process implemented on a CO_2_ laser splicer on a pre-existent grating. Moreover, the fact that the RI and temperature sensitivity can be separated into intensity-varying and spectral-shifting spectral features allows for an easy dual-parametric sensing system.

## 2. Materials and Methods

### 2.1. Fabrication of the stCFBG Fiber Sensor

The stCFBG has been designed by fabricating a shallow fiber taper in a pre-inscribed CFBG using a CO_2_ laser splicing system (Fujikura LZM100, Fujikura, Tokyo, Japan). The CFBG used in these experiments is a commercial device (Technica S.A.) having the following characteristics: bandwidth 15 nm (1562.5–1578.5 nm), length 15 mm, linear chirp with rate 1 nm/mm, and reflectivity ≥ 95% with <1 dB ripples in bandwidth. The CFBG was inscribed in a SMF (single-mode fiber, SMF-28 type).

The main adjustable parameters used for taper fabrication include waist diameter and taper length, with rotation mode switched off. After setting all the parameters, as provided in Table 1, CFBG was stripped using acetone, cleaned with isopropanol, and the region with the grating was placed between the holders in the laser operation area. When the “taper” button was pressed, the laser was activated and motors started to move at the set speed. As soon as the process was completed, a point-by-point measurement of newly fabricated taper was done with X and Y cameras in the equipment.

### 2.2. Gold Layer Sputtering

The stCFBG was prepped with Piranha solution (H_2_SO_4_ (70%) 3:1 (30%) H_2_O_2_) and sputtered with a 30-nm thin layer of Au (at a density of 19.32 g/m^3^, rate ranging from 3 to 7 nm/min, current 10 mA and pressure 1 × 10^−2^ mBar) on one side of the fiber surface with the sputter coater (Q150T Plus, Quorum Technologies Ltd., Lewes, UK). The g old-coated fiber was left to dry in the oven for two hours at 200 °C.

The gold thin film has been implemented in the stCFBG sensing structure for two purposes: in order to reduce the fiber losses in the tapered region, thus allowing a better spectral detection, and in order to mimic the functionalization process of a biosensor which makes use of metallic thin film to immobilize bioreceptors on the fiber surface.

### 2.3. Interrogation and Calibration

The stCFBG sensor has been interrogated using an optical backscatter reflectometer (OBR) interrogator (OBR46000, Luna Inc., Roanoke, VA, USA), using the following parameters: bandwidth 1525.0–1610.5 nm; resolution bandwidth 1.03 GHz; no electrical gain. The reflection spectra of the sensor have been digitally filtered using 7th order Chebyshev type-1 low-pass filter with a 0.014 digital frequency cut-off.

**Figure 1:**
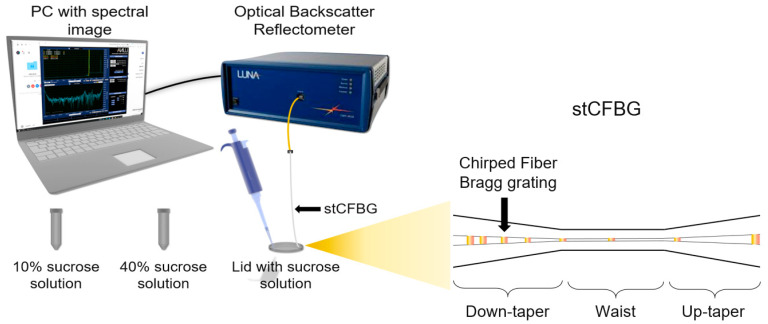
Schematic of the interrogation system—refractive index calibration.

Response to outer refractive index changes was measured using various concentrations of sucrose solution (Figure 1, 10% sucrose was used as a starting concentration to check the sensitivity of the gold-coated stCFBG. The concentration of the solution was increased as 40% sucrose was added gradually onto the lid, with the stCFBG inside changing the refractive index from 1.34974 to up to 1.35845.

**Figure 2:**
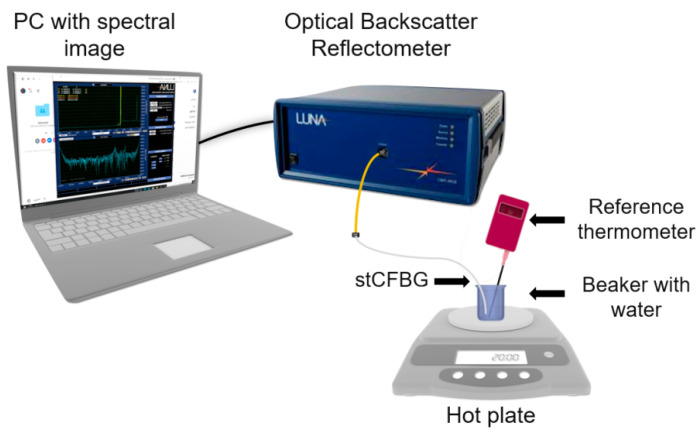
Schematic of the interrogation system—temperature calibration.

Temperature calibration of a gold-coated stCFBG submerged in a beaker with water maintaining a constant refractive index was performed on a hot plate (C-Mag HS4/IKA magnetic stirrer, IKA, Stausen, Germany), as shown in Figure 2, and a thermometer (ETS D-5/IKA electronic contact thermometer, IKA, Stausen, Germany) measuring the reference temperature was placed inside the same beaker. Spectral changes of gold-coated stCFBG were recorded every 5 °C as the temperature increased from 30 °C to 80 °C.

## 3. Results

### 3.1. Profilometry of the stCFBGs

**Figure 3:**
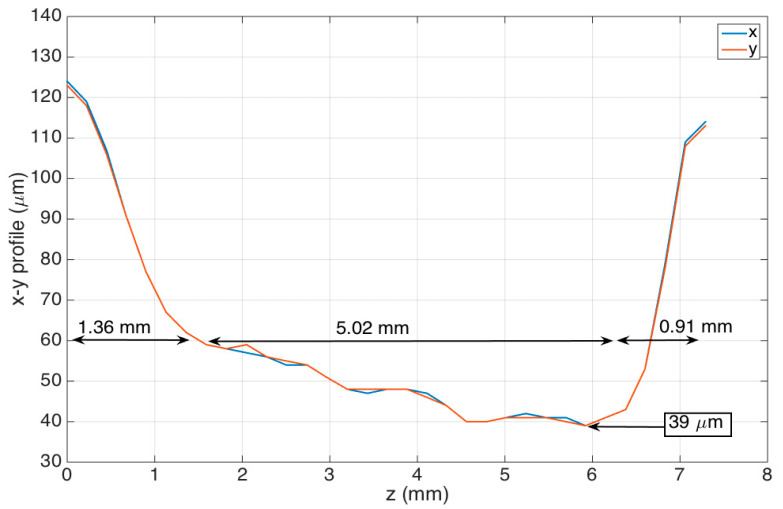
Profilometry of the fabricated stCFBGs, reporting the fiber thickness within the grating region as on x/y directions as a function of the fiber axis z.

In this section, we report the geometrical and spectral characteristics of the stCFBG presented in the previous section. Figure 3 shows the profilometry of the taper, as measured through the CO_2_ laser splicer; in this figure, we present the diameter along the horizontal axis x and vertical axis y (referred to the splicer, x along the splicer plane, y vertically aligned), as a function of the position along the fiber direction z.

The stCFBG device has a taper length that encompasses the whole region where the fiber is thinned of 7.29 mm. The taper has a left region, corresponding to the short CFBG wavelengths, with a length of 1.36 mm; the tapered slope, which defines the ratio of fiber thickness increase/reduction along z, is −46.3 μm/mm. The inner region, with a length of 5.02 mm, corresponds to the taper waist; here, the fiber progressively becomes thinner at a slower rate of −4.6 μm/mm, until it reaches the minimum taper waist of 39-μm thickness in correspondence to the 6-mm distance along z. The last region, corresponding to the output of the taper, shows a rapid increase of fiber thickness; it has a length of 0.91 mm and an average tapering slope of +82.4 μm/mm. In the thinner part of the waist region, the fiber shows a reduction of the thickness by a factor of 3.2, which accounts for a shallow taper; the V number in this region is estimated as 1.45 (according to the SMF-28 data for numerical aperture and core size), which shows how the fundamental mode is still confined within the core, and therefore, the taper modulates the fiber propagation losses as a function of the RI variation as main effect.

**Figure 4:**
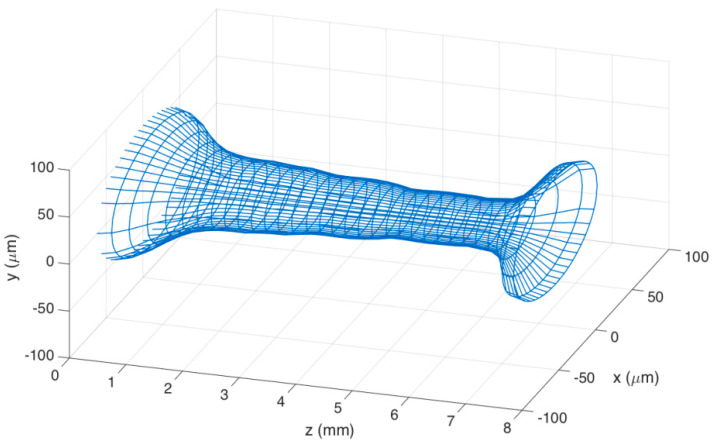
3D mesh of the fabricate taper.

Figure 4 shows the mesh of the stCFBG in the tapered region in a tri-dimensional chart, which allows visualization of the shape of the tapered region and the profile of the taper moving along the axis z. The mesh has been generated by projecting, for each fiber section along z, the elliptical profile on each xy plane having the diameters measured during the profilometry as x/y main axes.

### 3.2. Refractive Index Detection

**Figure 5:**
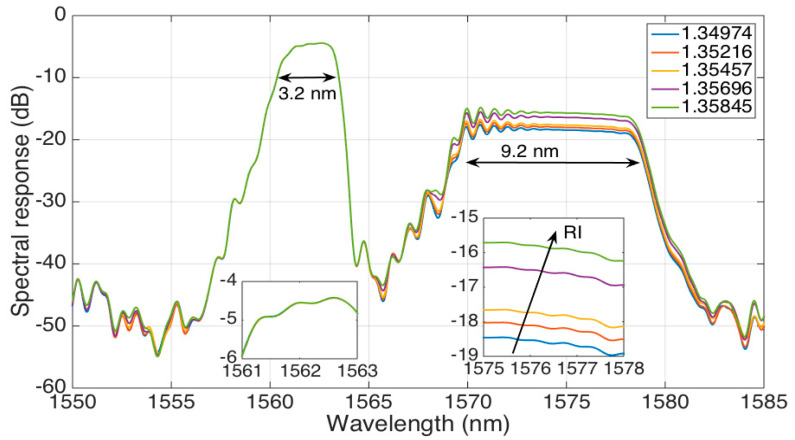
The reflection spectrum of the stCFBG, for five different RI values ranging from 1.34974 to 1.35845. The left inset shows the 1561–1563-nm bandwidth corresponding to the pre-taper zone, while the right inset shows the 1575–1578-nm bandwidth located after, in the post-taper region, which encodes the RI sensitivity.

The spectrum of the long stCFBG, for different values of RI ranging from 1.34974 to 1.35845, is shown in Figure 5. Here, we can observe that the device does not maintain the original shape of the CFBG with flat reflectivity across its reflection bandwidth, but rather, we can observe three separate spectral regions.

The leftmost part of the spectrum (3.2-nm wide), which corresponds to the shorter wavelengths, can be defined as a *pre-taper* region as it corresponds to the CFBG part lying before the tapered fiber section. Hence, the spectral characteristics of the CFBG are substantially maintained in this region, which shows a reflectivity level of −5 dB and a flat spectrum from 1561 nm to 1564 nm. As the light is perfectly confined in this section, the spectral characteristics are RI-independent, as shown in the left inset of Figure 5.

The inner part of the spectrum, from 1564 nm to 1570 nm, corresponds to the tapered part of the stCFBG. Here, the increase of evanescent waves in correspondence to this wavelength range causes a significant reduction of the reflectivity level down to −45 dB.

Finally, the rightmost part of the spectrum (9.2-nm wide), covering 1570–1579 nm, can be labeled as the *post*-taper region: it corresponds to the stCFBG part located at the end side of the taper, and therefore, the light reflected in this region runs twice (forward and backward) across the tapered part. As shown in [21], the taper losses are RI-dependent as the light is reflected at an interface between the cladding and the surrounding environment with different reflection coefficients. In this region, the RI sensitivity is evident, as the spectral level increases by 2.6 dB for a RI change of 0.0087; this effect is shown in the right inset of the figure, which shows how the RI change converts into a reflectivity level variation. Due to the inline propagation losses, the reflectivity level for this range floats around 12–14 dB lower than the pre-taper level.

Hence, the spectral fingerprint of the stCFBG allows an easy identification of the RI sensitivity, as it is simply sufficient to detect the spectral intensity change within the post-taper bandwidth, either at a single wavelength or across the whole bandwidth for a more accurate response.

### 3.3. Temperature Detection

The temperature sensitivity of the stCFBG follows the thermal response of a generic CFBG [25], which is similar to any standard FBG [26]: as the local Bragg wavelength reflected at any point of the grating changes, with a linear coefficient, due to temperature changes, we expect a shift of the grating spectrum when the temperature experiences a change.

**Figure 6:**
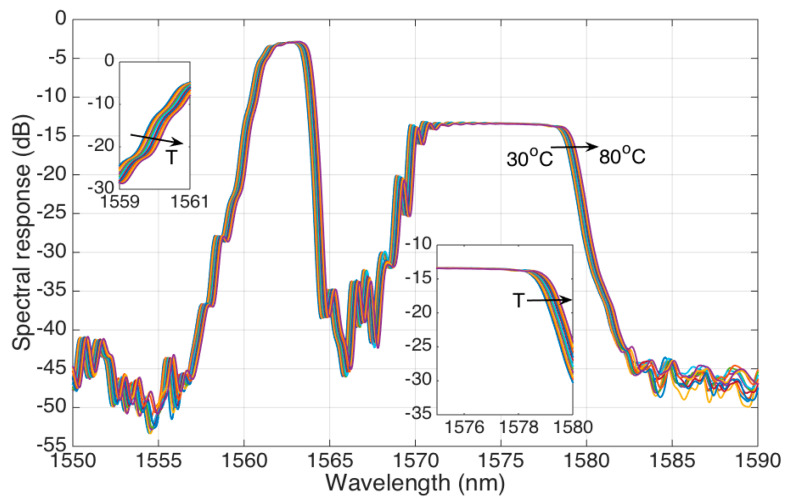
The reflection spectrum of the stCFBG, in water, for temperature values ranging from 30 °C to 80 °C. The left inset shows the response on the left side of the grating spectrum (1559–1561 nm) where we observe the wavelength shift; the right inset shows the response on the 1575–1580 nm bandwidth, where we observe the insensitivity of the reflectivity level to the temperature, as well as the shift of the whole spectral bandwidth towards the longer wavelengths.

This trend is observed in Figure 6, where the spectrum of the stCFBG in water is shown for temperature values rising from 30 °C to 80 °C in steps of 5 °C. We observe that the temperature change results in a much different behavior than the RI change. The whole spectral fingerprint shifts towards the longer wavelengths, proportionally to the temperature increase; this effect can be shown on each region of the stCFBG, including the left side and right side of the pre-taper region (first inset of Figure 6), and the left and right sides of the post-taper region, as in the second inset. However, the level of reflectivity does not change through the spectrum, as both the pre-taper and post-taper levels are almost constant when the temperature changes.

### 3.4. Sensitivity Analysis and Dual RI/Temperature Sensing

The stCFBG device allows a dual RI/temperature sensing, as the two physical changes affect different features of the spectral fingerprint. The variation of RI from a reference value, hereby labeled Δn, is detected by measuring the change of intensity of the reflection spectrum (ΔI), integrated over the post-taper region. On the other side, the temperature variation (ΔT) from a reference value is measured by evaluating the wavelength shift (Δλ) of the pre-taper bandwidth, estimated by performing the spectral cross-correlation between the reference and measured spectra [25].

**Figure 7:**
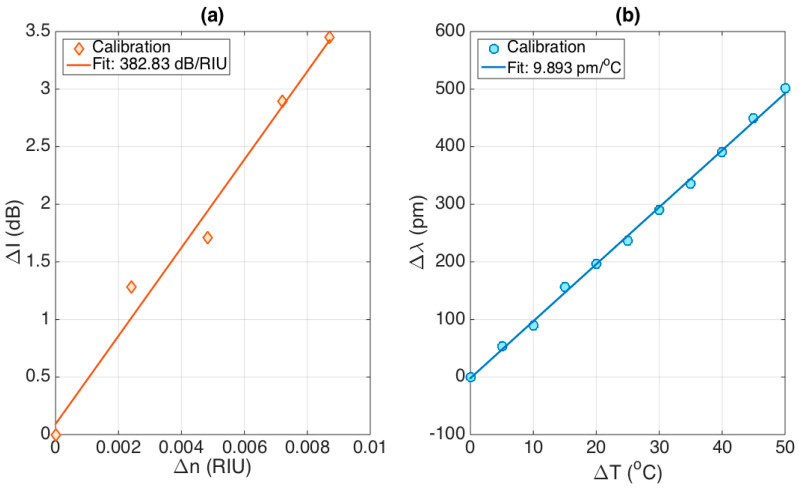
Evaluation of the dual stCFBG sensitivity to RI and temperature. (**a**) RI response, evaluating ΔI as a function of Δ*n*; the sensitivity ΔI/Δ*n* is estimated as 382.83 dB/RIU, with R^2^ = 0.9822. (**b**) Temperature response, evaluating Δλ as a function of ΔT; the sensitivity Δλ/ΔT is estimated as 9.893 pm/°C, with R^2^ = 0.9982.

The results of the sensitivity analysis are shown in Figure 7. The left chart shows the RI sensitivity, which shows a trend that has good linearity over the working range. In order to mimic a typical application in biosensors, which is facilitated by the presence of a gold film coating the fiber [6], the calibration has been performed over a short working range (8.7 × 10^−3^ RIU). The linear fit (R^2^ = 0.9822) allows estimating the RI sensitivity as ΔI/Δ*n* = 382.83 dB/RIU. The second chart shows the temperature calibration, performed over a 50 °C interval. The data show great linearity, similar to the typical operation of FBGs, with a sensitivity Δλ/ΔT = 9.893 pm/°C (R^2^ = 0.9982), which is a coefficient similar to the nominal ~10 pm/°C for FBGs inscribed into glass fibers [26].

**Figure 8:**
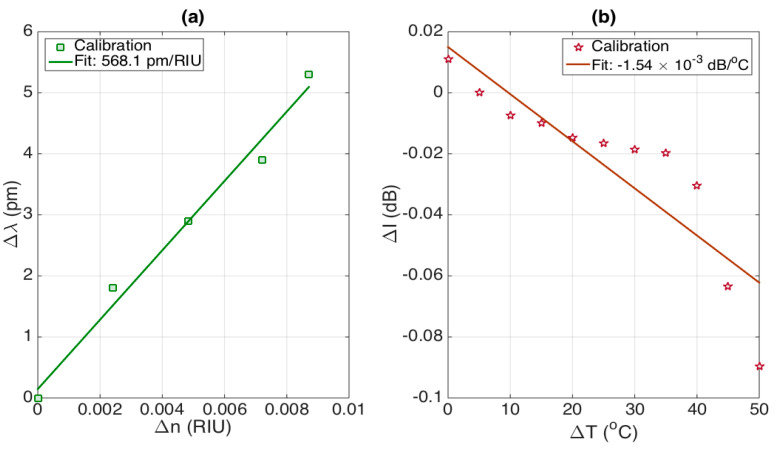
Evaluation of the cross-sensitivities of the stCFBG sensitivity to RI and temperature. (**a**) Effect of the RI change on the wavelength shift estimation, evaluated as 568.1 pm/RIU; (**b**) effect of the temperature change on the intensity level of the post-taper bandwidth, estimated as −1.54 × 10^−3^ dB/°C.

In Figure 8, we evaluate the cross-sensitivities, i.e., Δλ/Δ*n* and ΔI/ΔT; unlike several dual RI-temperature sensors, the stCFBG exhibits small cross-sensitivities. The first chart reports the wavelength shift as a function of RI change, which shows an almost linear pattern (R^2^ = 0.9839) and a slope of 568.1 pm/RIU. On the second chart, we report the intensity level change of the post-taper region as a function of the temperature change. The effect is smooth until the temperature reaches ~70 °C, and after this value, it shows a drop which, however, is limited to fractions of dB. The average cross-sensitivity is estimated as −1.54 × 10^−3^ dB/°C over this range (R^2^ = 0.7843).

As a benchmark, we can consider the effect of a temperature variation of 1 °C on the RI estimate, which would lead to a change of 1.54 × 10^−3^ dB, corresponding to 4.0 × 10^−6^ RIU detuning. Similarly, a change of 10^−3^ RIU would lead to a wavelength shift change of 0.57 pm, which corresponds to a detuning term of 0.057 °C. Overall, we can affirm that cross-sensitivity effects on small changes of RI and temperature are substantially negligible, and therefore, the stCFBG sensing unit behaves as an almost perfectly separated detector of RI and temperature.

In the alternative, dual-parametric sensing can be implemented in a matrix-based method [27] that also takes into account the cross-sensitivities:

(1) $$\left[\begin{array}{c} \Delta I\\ \Delta \lambda \end{array}\right]=\left[\begin{array}{cc} 382.83\;\mathrm{dB}/\mathrm{RIU} & -1.54\times 10^{-3}\;\mathrm{dB}/{}^{\circ}C\\ 568.1\;\mathrm{pm}/\mathrm{RIU} & 9.893\;\mathrm{pm}/{}^{\circ}C \end{array}\right]\left[\begin{array}{c} \Delta n\\ \Delta T \end{array}\right]$$

where the elements on the main diagonal contain the sensitivity values, and the other two elements contain the cross-sensitivity terms. As proof of the validity of the low cross-sensitivity, the product of the elements on the main diagonal is 3787.3 dB pm RIU^−1^. °C^−1^, while the product of the other two elements is 0.9 dB pm RIU^−1^ °C^−1^, which is significantly smaller. The system in Equation (1) can be resolved by inverting the inner matrix:

(2) $$\left[\begin{array}{c} \Delta n\\ \Delta T \end{array}\right]=\left[\begin{array}{cc} 2.611\times 10^{-3}\;\mathrm{RIU}/\mathrm{dB} & -4.065\times 10^{-7}\;\mathrm{RIU}/\mathrm{pm}\\ -0.1499\;\mathrm{RIU}/\mathrm{pm}, & 0.1011\;{}^{\circ}C/\mathrm{pm} \end{array}\right]\left[\begin{array}{c} \Delta I\\ \Delta \lambda \end{array}\right]$$

## 4. Discussion

The results hereby achieved for the simultaneous detection of RI and temperature on a gold-coated stCFBG provide a promising tool for a robust biosensing technology, capable of working in situ as a long-term monitoring unit (for example, for continuous monitoring of analyte over the long term [2]) or for interaction with biological films that require a continuous detection system [28]. The gold coating is an effective method for immobilizing bioreceptors, and therefore, this structure can be converted from a compensated refractometer to a biosensor through a specific biofunctionalization process [6].

The first advantage of the proposed method relies on the robustness and ease of fabrication and interrogation, which makes it a repeatable and scalable method for the mass-manufacturing of sensors. The CFBG unit is pre-inscribed in the fiber, and therefore, the sensor can be directly manufactured on a commercial chirped grating (which is very common for sensing or dispersion compensation in telecommunications); the taper fabrication is a single-step process with a commercial splicer, which is simply derived from methods used in optical tweezers or fiber adapters, as opposite to Fabry–Perot interferometers based on cascades of tapers that require more artisanal and cumbersome fusion methods [29]. Most notably, the sensor is capable to express a high sensitivity, higher than tilted FBGs [13] or U-bent fibers [30] in terms of intensity change per RIU, even with a shallow taper, with a 39-μm waist size. In general, tapered fibers have a diameter in the order of few micrometers or below, in order to obtain interferometric effects [24], which, however, makes them vulnerable to mechanical stress and, therefore, extremely fragile when put in operation. Conversely, the stCFBG appears as a robust sensing unit, as the fiber maintains high tensile strength and suitability to be packaged in a medical device.

The second advantage is in the capability to break down the RI and temperature sensing capabilities into two distinct spectral features, with minimal cross-talk, as we can split the bandwidth in which the features are analyzed. Table 2 shows the performance of the proposed method, compared with other dual-parametric RI/temperature fiber optic sensing methods.

Interferometric methods are commonly used for RI/temperature dual-sensing, as they allow the detection of multiple spectral dips. Wang et al. reported a dual-cavity interferometric method [31] which is optimized for gas sensing, while Ran et al. [17] reported a Bragg grating in a microfiber that achieves cardiac biomarker detection with a low limit of detection. This method in particular shows good sensitivity, but the cross-sensitivity values (10.49 nm/RIU and 5.73 pm/°C) require a full analysis of the sensitivities, similar to Equation (2), in order to correctly identify each parameter. Similarly, Huang et al. [32] optimized the sensitivity of a Fabry–Perot interferometer achieving 1591 nm/RIU sensitivity and relatively small temperature sensitivity (1.02 nm/°C). SPR is another method for dual-parametric sensing that has found several applications: among others, Velázquez-González et al. [33] reported a SPR-based method based on a modal interferometer that achieves a simultaneous high sensitivity (2323 nm/RIU) and low crosstalk (0.28 nm/°C) by tracking two separate spectral features. Pevec and Donlagic [16] proposed a micromachined Fabry–Perot interferometer with dual sensitivity that has a quite simple fabrication process.

Long-period gratings (LPGs) represent the main grating-based technology for dual sensing. Fan et al. [34] reported a hybrid LPG with an integrated TFBG for dual-parametric sensing, which requires a full matrix-like analysis, as both the TFBG and the LPG are RI and temperature-sensitive, with different coefficients. Esposito et al. [15] reported LPG-based method for three-parameter analysis (RI, temperature, strain), based on the joint analysis of multiple spectral peaks and dips. Similarly, Li et al. [27] proposed an ambitious structure based on a 4-core fiber with an FBG, in order to obtain a simultaneous RI, temperature, and strain sensing through the analysis of two spectral dips and the Bragg wavelength shift. For these works, Table 2 presents the maxima for sensitivity values and minima for cross-sensitivity, although a full 3 × 3 matrix analysis is needed for the multi-parametric estimate.

Overall, the proposed work improves the state of the art in terms of fabrication simplicity, low cross-talk, and the possibility to operate the sensor on a narrow bandwidth (about 20 nm in this work, in comparison to the hundreds of nanometers for interferometers and LPGs [35,36,37].

## 5. Conclusions

In conclusion, we reported the fabrication and interrogation of a gold-coated shallow-tapered chirped FBG, for dual-parametric RI and temperature sensing. The fabrication is simply based on a shallow and compact fiber taper (39 μm waist size, 7.29 mm length) obtained by a CO_2_ laser splicer manufacturing on a pre-inscribed chirped FBG. The spectral analysis shows a pre-taper region, insensitive to RI and where the temperature sensitivity can be estimated by wavelength shift detection, and a post-taper region in which the reflectivity level varies with the RI.

The sensitivity of the device is estimated as 382.83 dB/RIU for RI detection and 9.893 pm/°C; the cross-sensitivity values are low, respectively −1.54 × 10^−3^ dB/°C and 568.1 pm/RIU, which allows the sensor to operate with minimal cross-talk.

Thanks to the dual sensitivity, the compact size, the shallow taper structure that allows the fiber to maintain its mechanical strength, and the gold-coating that allows the immobilization of bioreceptors, the stCFBG structure can be a promising technology for the realization of fiber-optic biosensors for in situ analysis and long-term monitoring.

## Figures and Tables

**Table 1:** Input parameters for fabrication of shallow-tapered chirped fiber Bragg grating.

Fabrication Parameter	Value
Initial fiber diameter (μm)	125
Waist diameter (μm)	30
Left taper length (mm)	0.5
Waist taper length (mm)	5
Right taper length (mm)	0.5
Pre-heat (bit)	0
Absolute power (bit)	632
Relative power (bit)	100
Waist add (bit)	20
Pulling speed (mm/sec)	0.18

**Table 2:** Comparison of the main methods for dual RI/temperature fiber optic sensing, outlining the main performance metrics.

Reference	Sensing Method	RI Sensitivity	Temperature Sensitivity	Cross-Sensitivities
Wang et al. [31]	Dual-cavity Fabry–Perot interferometer	1536 nm/RIU	80.7 pm/°C	
Ran et al. [17]	Micro-fiber Bragg grating	59.56 nm/RIU	3.56 pm/°C	10.49 nm/RIU 5.73 pm/°C
Huang et al. [32]	Graphene-coated modal interferometer	1591 nm/RIU	1.02 nm/°C	
Velázquez-González et al. [33]	SPR modal interferometer	2323 nm/RIU	2.85 nm/°C	0.28 nm/°C
Pevec et al. [16]	Micromachined Fabry–Perot	1067 nm/RIU	9.87 pm/°C	
Fan et al. [34]	Hybrid grating LPG/TFBG	606.82 nm/RIU	268.8 pm/°C	
Esposito et al. [15]	LPG inscribed in Panda fiber	12.1 nm/RIU	79.1 pm/°C	0.7 nm/RIU 15.5 pm/°C
Li et al. [27]	Four-core fiber with FBG	106.2 nm/RIU	194.6 pm/°C	9.07 pm/°C ~0 nm/RIU
This work	Shallow-tapered chirped FBG	382.8 dB/RIU	9.89 pm/°C	568 pm/RIU 1.5 × 10^−3^ dB/°C

## Data Availability

Not applicable.
